# Supplementary material for: Statistical power in COVID-19 case-control host genomic study design
Source: Genome Med. 2020 Dec 28;12:115. doi: 10.1186/s13073-020-00818-2 (PMC7768597; doi:10.1186/s13073-020-00818-2)
Supplement: Supplementary file 1 — Additional file 1: Supplementary Methods. Detailed simulation settings for studying SARS-CoV-2 Infection Susceptibility. [file 13073_2020_818_MOESM1_ESM.docx]

**Simulation Settings: SARS-CoV-2 Infection Susceptibility**

Variables

- Exposure to SARS-CoV-2 ($Exposure$)
  - Binary. “1” for exposed and “0” for unexposed.
- SARS-CoV-2 Infection ($Infection$)
  - Binary. “1” for infected and “0” for uninfected.
- COVID-19 associated hospitalization ($Hospitalization$)
  - Binary. “1” for hospitalized and “0” for non-hospitalized/mild symptoms.
- Received testing for SARS-CoV-2 infection or not ($If\_tested$)
  - Binary. “1” if the person received testing and “0” otherwise.
- Test result for SARS-CoV-2 infection ($Test\_result$)
  - Binary. “1” if tested positive and “0” if tested negative.
- Number of protection alleles carried: Variant associated with infection susceptibility
  - $G_{inf}$; 0, 1, or 2
- Number of risk alleles carried: Variant associated with disease severity / hospitalization
  - $G_{hosp}$; 0, 1, or 2

Parameters

- Population Exposure Rate ($p_{exposure}$)
- Minor allele frequency of $G_{inf}$ ($MAF_{inf}$)
- Minor allele frequency of $G_{hosp}$ ($MAF_{hosp}$)
- Baseline Infection Susceptibility ($p_{suscep}$)
  - Probability of infection on exposure to SARS-CoV-2 in the absence of the contributing protective genetic allele.
- Baseline risk of hospitalization ($p_{hosp}$)
  - Probability of developing severe symptoms/hospitalization given SARS-CoV-2 infection in the absence of the contributing risk allele.
- Test prevalence for individuals with SARS-CoV-2 infection and mild symptoms
  - $p_{test\_prev\_mild}$
  - Test prevalence for individuals with severe symptoms is assumed to be 100%
- Test prevalence for individuals without SARS-CoV-2 infection

- - $p_{test\_prev\_no\_inf}$
- Decreased risk of infection with an additional protection allele of $G_{inf}$
  - $OR_{inf}$; Log-additive.
- Increased risk of hospitalization with an additional risk allele of $G_{hosp}$
  - $OR_{hosp}$; Log-additive
- Test sensitivity $(sens)$
- Test specificity $(spec)$

Simulated Data Generating Mechanism

1. **Simulate genetic variants.** Each individual $i$ carries
   1. Variant (Infection Susceptibility): $G_{inf,i}\sim Binom\left( 2, MAF_{inf} \right)$.
   2. Variant (Disease Severity / Hospitalization): $G_{hosp,i}\sim Binom(2, MAF_{hosp})$
2. **Simulate exposure to SARS-CoV-2.**
   1. $Exposure_{i}\sim Bernoulli(p_{exposure})$
3. **Simulate SARS-CoV-2 infection.**
   1. If $Exposure_{i}=0$, $Infection_{i}=0.$
   2. If $Exposure_{i}=1$ and $G_{inf,i}=0$, $Infection_{i}\sim Bernoulli(p_{suscep})$
   3. If $Exposure_{i}=1$ and $G_{inf,i}\neq0$, $Infection_{i}\sim Bernoulli\left( f\left( f^{-1}\left( p_{suscep} \right)+G_{inf,i}\times OR_{inf} \right) \right)$, where $f$ is the logistic function.
4. **Simulate severe symptoms / hospitalization given SARS-CoV-2 infection.**
   1. If $Infection_{i}=0$, $Hospitalization_{i}=0$.
   2. If $Infection_{i}=1$ and $G_{hosp,i}=0$, $Hospitalization_{i}=Bernoulli(p_{hosp})$
   3. If $Infection_{i}=1$ and $G_{hosp,i}\neq0$, $Hospitalization_{i}\sim Bernoulli\left( f\left( f^{-1}\left( p_{hosp} \right)+G_{hosp,i}\times OR_{hosp} \right) \right)$ where $f$ is the logistic function.
5. **Simulate whether the individual received testing for SARS-CoV-2 infection.**
   1. If $Infection_{i}=0$, $If\_tested_{i}\sim Bernoulli(p_{test\_prev\_no\_inf})$
   2. If $Infection_{i}=1$ and $Hospitalization_{i}=0$, $If\_tested_{i}\sim Bernoulli(p_{test\_prev\_mild})$
   3. If $Hospitalization_{i}=1,$ $If\_tested_{i}=1$
6. **Simulate Test Result for SARS-CoV-2 infection.**
   1. If $Infection_{i}=0$, $Tes{t\_result}_{i}\sim Bernoulli(1-spec)$
   2. If $Infection_{i}=1$ and $Hospitalization_{i}=0$, $Tes{t\_result}_{i}\sim Bernoulli(sens)$
   3. If $Hospitalization_{i}=1$, $Tes{t\_result}_{i}=1$

Infection Susceptibility Study Design

**Inclusion Criteria**

1. Those who received testing for SARS-CoV-2 infection $(I{f\_tested}_{i}=1)$.

**Case-control definition**

1. Cases: Test-positive individuals $(Te{st\_result}_{i}=1)$.
2. Controls: Test-negative individuals $(Tes{t\_result}_{i}=0)$.

Standard univariate logistic regression was used to estimate the effect size of the genetic variant. A finding is reported if the p-value is below the genome-wide significance threshold, 5e-8.

Default Parameter Settings

1. Baseline Infection Susceptibility ($p_{suscep}$)

- 80% given current estimates for SARS-CoV-2 infection [11].

1. Baseline risk of hospitalization ($p_{hosp}$)

- 5%. States/Provinces across U.S. and Canada currently report hospitalization rates between 8~10% but may overestimate the figure due to many asymptomatic patients / false negatives.

1. Test prevalence for individuals with SARS-CoV-2 infection and mild symptoms

- $p_{test\_prev\_mild}$
- 30%

1. Test prevalence for individuals without SARS-CoV-2 infection

- $p_{test\_prev\_no\_inf}$
- 5%

Case-Control Misclassification

Proportion of population received testing and tested positive for SARS-CoV-2 infection

$$total\_cases=p_{exposure}\times p_{suscep}\times\left( p_{hosp}\times1+\left( 1-p_{hosp} \right)\times p_{test\_prev\_mild} \right)\times sens+\left( \left( 1-p_{exposure} \right)+p_{exposure}\times\left( 1-p_{suscep} \right) \right)\times p_{test\_prev\_no\_inf}\times(1-spec)$$

Proportion of population received testing and tested negative for SARS-CoV-2 infection

$$total\_controls=p_{exposure}\times p_{suscep}\times\left( p_{hosp}\times1+\left( 1-p_{hosp} \right)\times p_{test\_prev\_mild} \right)\times(1-sens)+\left( \left( 1-p_{exposure} \right)+p_{exposure}\times\left( 1-p_{suscep} \right) \right)\times p_{test\_prev\_no\_inf}\times spec$$

| **Misclassification among cases** |  |
| --- | --- |
| True controls (uninfected) misclassified as cases due to false positives produced by RT-PCR tests | $\frac{\left( 1-p_{exposure} \right)\times p_{test\_prev\_no\_inf}\times(1-spec)}{total\_cases}$ |
| **Misclassification among controls** |  |
| Unexposed individuals that should have been infected upon exposure | $\frac{\left( 1-p_{exposure} \right)\times p_{test\_prev\_no\_inf}\times p_{suscep}}{total\_controls}$ |
| True cases (infected) misclassified as controls (uninfected) due to a low sensitivity RT-PCR test | $\frac{p_{exposure}\times p_{suscep}\times(1-p_{hosp})\times p_{test\_prev\_mild}\times(1-sens)}{total\_controls}$ |
