# Supplementary material for: Statistical power in COVID-19 case-control host genomic study design
Source: Genome Med. 2020 Dec 28;12:115. doi: 10.1186/s13073-020-00818-2 (PMC7768597; doi:10.1186/s13073-020-00818-2)
Supplement: Supplementary file 2 — Additional file 2: Supplementary Figures and Tables. Figure S1. Statistical power to detect associations between genetic variants and infection susceptibility at the genome-wide significance level (5e-8) when the test sensitivity is low (sensitivity = 0.7). Figure S2. Statistical power to detect a true association between a genetic variant and COVID-19 disease severity at the genome-wide significance level (5e-8). Figure S3. Statistical power to detect a true association between a genetic variant and COVID-19 disease severity at the genome-wide significance level (5e-8) when varying the case-control ratio. Table S1. Relative reduction in sample size, $1-\frac{n_{test\_ positive\_ controls}}{n_{population\_ controls}}$1−ntest_positive_controlsnpopulation_controls, from using test-positive controls compared to population-based controls. [file 13073_2020_818_MOESM2_ESM.docx]

**Supplementary Figures and Tables**


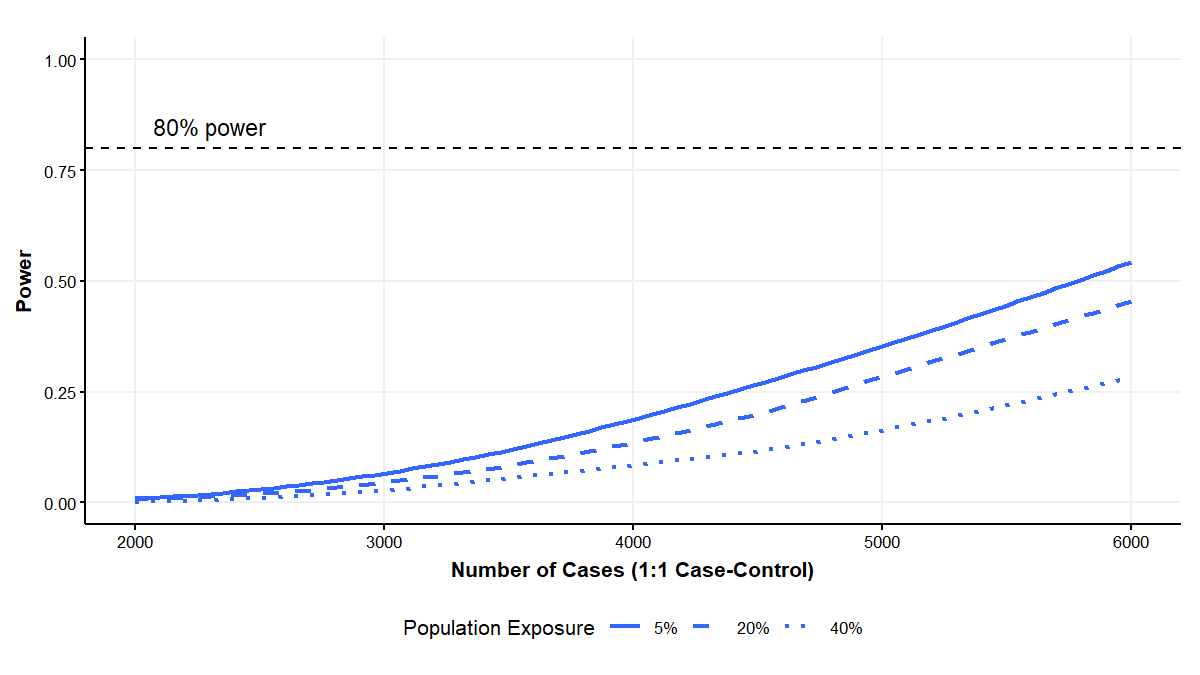


**Figure S1:** **Statistical power to detect associations between genetic variants and infection susceptibility at the genome-wide significance level (5e-8) when the test sensitivity is low (sensitivity = 0.7).** A 1:1 case-control study design was used for all parameter settings. The plot assumes a common variant with large effect size (OR=0.5, MAF=0.2). Reducing sensitivity for testing SARS-CoV-2 infection not only reduces statistical power but also negates gains that result from increasing population exposure. In fact, increasing population exposure leads to reduced statistical power when the test sensitivity is too low.


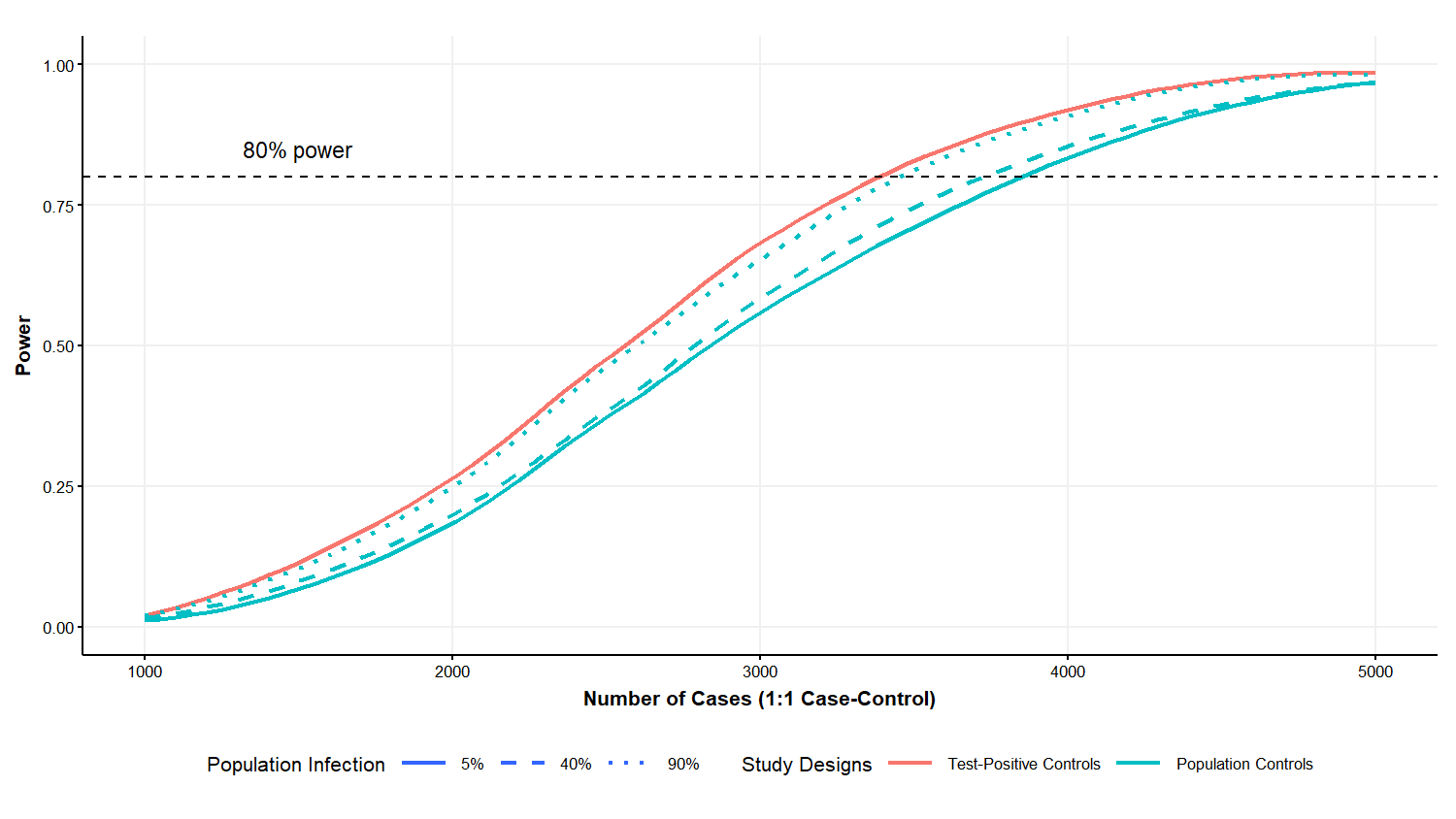


**Figure S2:** **Statistical power to detect a true association between a genetic variant and COVID-19 disease severity at the genome-wide significance level (5e-8).** A 1:1 case-control study design was used for all parameter settings. Only one red curve is shown since the study design uses confirmed infected individuals with mild or no symptoms as controls (test-positive controls), which is unaffected by population-level infection rates and the corresponding case-control misclassification. Effect sizes are reported on the odds ratio (OR) scale for each additional risk allele. Assumes perfect test accuracy. Detecting a common variant with moderate effect size (OR=1.3, MAF=0.2) is much more challenging without drastically increasing the number of participants studied. In fact, the sample size required to reach 80% power exceeds 4 times that required when the effect size is large (OR=1.7).

**Table S1:** **Relative reduction in sample size,** $\boldsymbol{1-}\frac{\boldsymbol{n}_{\boldsymbol{test\_positive\_controls}}}{\boldsymbol{n}_{\boldsymbol{population\_controls}}}$**, from using test-positive controls compared to population-based controls.** $n_{test\_positive\_controls}$ and $n_{population\_controls}$ refer to the number of cases (1:1 case-control ratio) needed to achieve 80% power at the genome-wide significance level (5e-8) [22]. Using controls with confirmed infection show the greatest benefit when disease prevalence is low. Given current levels of population infection rates, choosing controls with confirmed SARS-CoV-2 infection can save over 10% in genotyping costs.


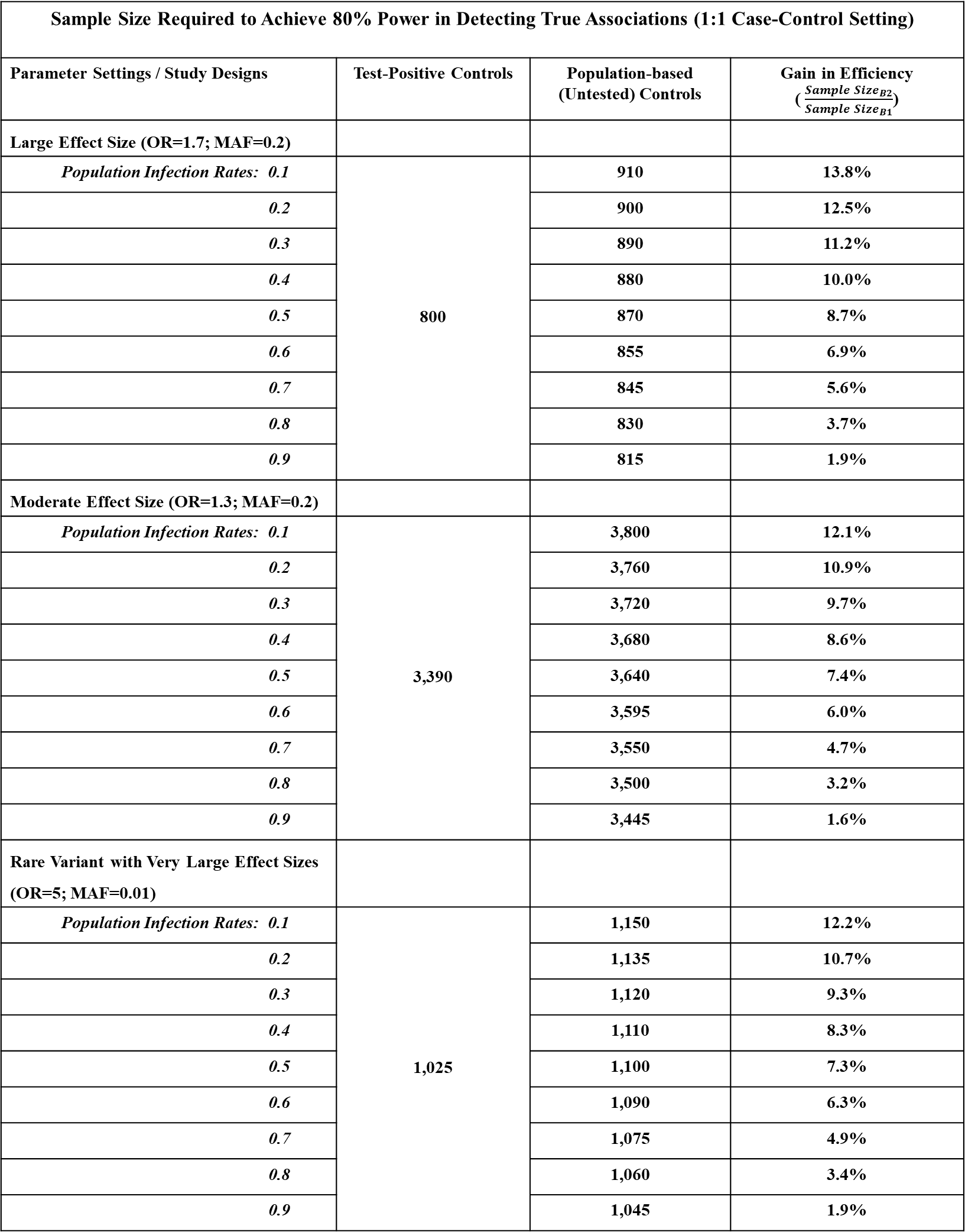


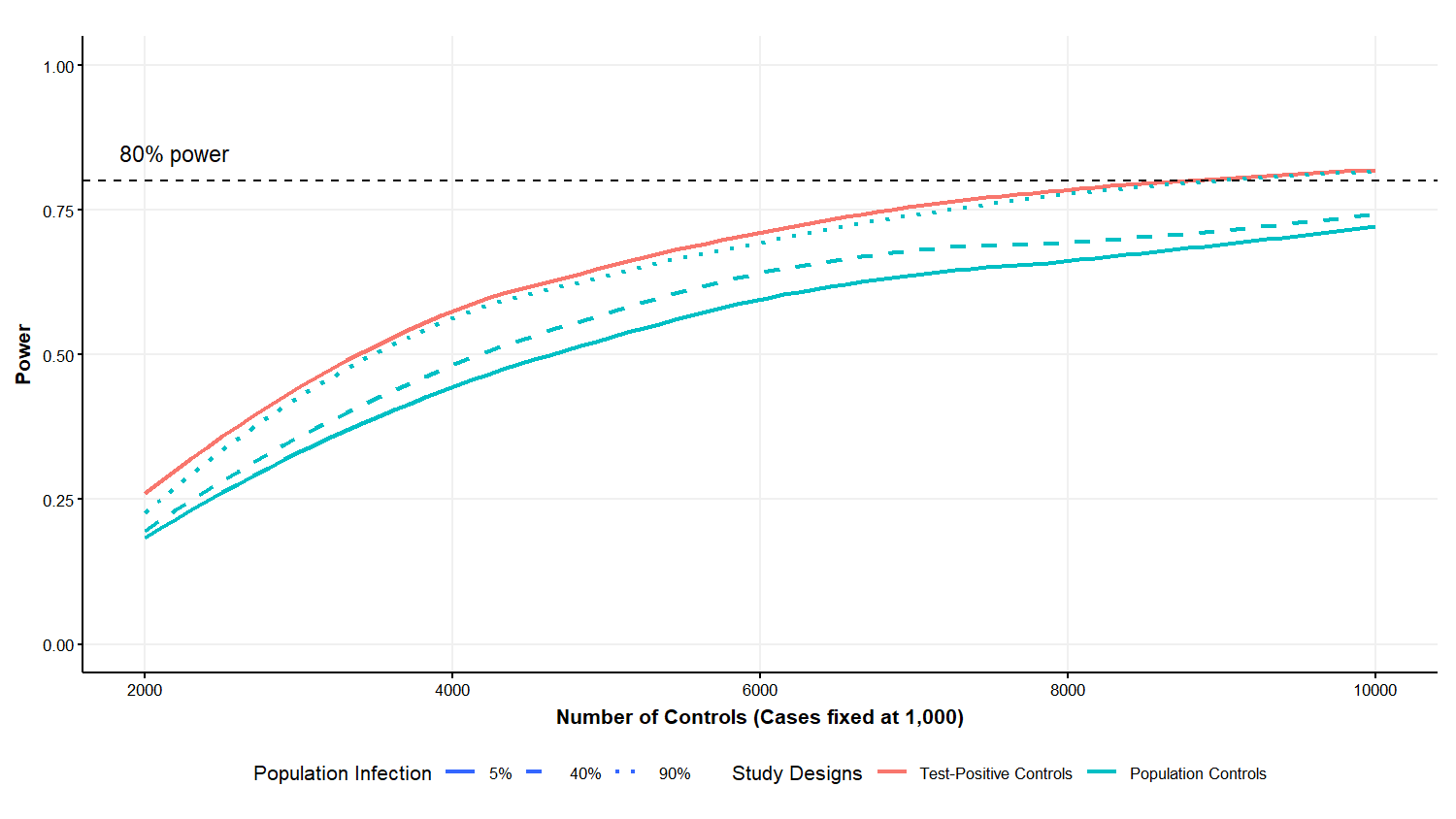


**Figure S3:** **Statistical power to detect a true association between a genetic variant and COVID-19 disease severity at the genome-wide significance level (5e-8) when varying the case-control ratio.** Cases are fixed at 1,000 and controls are varied to allow reduced case-control ratio. Effect sizes are reported on the odds ratio (OR) scale for each additional risk allele. Assumes perfect test accuracy. Detecting a common variant with moderate effect size (OR=1.3, MAF=0.2) requires much larger sample sizes if a reduced case-control ratio is used.
