# Supplementary material for: Statistical power in COVID-19 case-control host genomic study design
Source: Genome Med. 2020 Dec 28;12:115. doi: 10.1186/s13073-020-00818-2 (PMC7768597; doi:10.1186/s13073-020-00818-2)
Supplement: Supplementary file 3 — Additional file 3: Supplementary Methods. Detailed simulation settings for studying COVID-19 disease severity. [file 13073_2020_818_MOESM3_ESM.docx]

**Simulation Settings: COVID-19 Disease Severity**

Variables, parameters and the simulation process are all identical to those provided in Additional File 1.

Disease Severity Study Design

**Using Test-Positive Controls**

**Inclusion Criteria**

1. Those who received testing for SARS-CoV-2 infection and tested positive $(If\_tested_{i}=1 \& tes{t\_result}_{i}=1)$.

**Case-control definition**

1. Cases: Individuals with severe symptoms (hospitalized; $Hospitalization_{i}=1$).
2. Controls: Individuals without severe symptoms ($Hospitalization_{i}=0$).

**Using Population-based (untested) Controls**

**Inclusion Criteria**

1. Those who received SARS-CoV-2 infection, tested positive and were hospitalized ($Hospitalization_{i}=1$), and the population-based controls that never received testing ($If\_tested_{i}=0$).

**Case-control definition**

1. Cases: Individuals with severe symptoms (hospitalized; $Hospitalization_{i}=1$).
2. Controls: Population-based controls that never received testing ($If\_tested_{i}=0$).

Standard univariate logistic regression was used to estimate the effect size of the genetic variant. A finding is reported if the p-value is below the genome-wide significance threshold, 5e-8.

Default Parameter Settings

Contrary to the low population exposure that drives case-control misclassification in SARS-CoV-2 infection susceptibility studies, it is the low population *infection* rates and the lack of information on individual-level *infection* that can lead to misclassification in disease severity studies; as some of the defined “controls” would have developed severe symptoms had they been infected with SARS-CoV-2.

1. Baseline Infection Susceptibility ($p_{suscep}$)

- 100%. Since it is the low population *infection* rates and the lack of information on individual-level *infection* that can lead to misclassification in disease severity studies. Varying population exposure is equivalent to varying population infection rates if $p_{suscep}=1$.

1. Baseline risk of hospitalization ($p_{hosp}$)

- 5%. States/Provinces across U.S. and Canada currently report hospitalization rates between 8~10% but may overestimate the figure due to many asymptomatic patients / false negatives.

1. Test prevalence for individuals with SARS-CoV-2 infection and mild symptoms

- $p_{test\_prev\_mild}$
- 30%

1. Test prevalence for individuals without SARS-CoV-2 infection

- $p_{test\_prev\_no\_inf}$
- 5%.

Case-Control Misclassification

Define population infection rate as $p_{inf}=p_{exposure}\times p_{suscep}$.

**Using population-based (uninfected) controls**

| Misclassification of controls |  |
| --- | --- |
| Uninfected individuals that should have been hospitalized upon infection | $(1-p_{inf})\times p_{hosp}$ |

**Using test-positive controls**

| Misclassification of controls |  |
| --- | --- |
| Test-positive controls (infected) which were in fact not infected due to false positives produced by RT-PCR tests. Some would have developed severe symptoms upon infection. | $\frac{\left( 1-p_{inf} \right)\times(1-spec)}{p_{inf}\times sens+\left( 1-p_{inf} \right)\times(1-spec)}\times p_{hosp}$ |
